# Quantitative PCR as a marker for preemptive therapy and its role in therapeutic control in *Trypanosoma cruzi*/HIV coinfection

**DOI:** 10.1371/journal.pntd.0011961

**Published:** 2024-02-26

**Authors:** Vera Lúcia Teixeira de Freitas, Christina Terra Gallafrio Novaes, Ana Marli Christovam Sartori, Noemia Barbosa Carvalho, Sheila Cristina Vicente da Silva, Érika Shimoda Nakanishi, Fernando Salvador, Cleudson Nery de Castro, Rita Cristina Bezerra, Elizabeth Visone Nunes Westphalen, Caroline Medeji Ramos de Oliveira, Felipe Delatorre Busser, Yeh-Li Ho, Renata Buccheri, Carolina Bonilla, Maria Aparecida Shikanai-Yasuda

**Affiliations:** 1 Departamento de Molestias Infecciosas e Parasitarias, Faculdade de Medicina, Universidade de Sao Paulo, São Paulo, São Paulo, Brazil; 2 Laboratorio de Investigacao Medica em Imunologia (LIM 48), Hospital das Clinicas, Faculdade de Medicina, Universidade de Sao Paulo, São Paulo, Brazil; 3 Divisao de Molestias Infecciosas e Parasitarias, Hospital das Clinicas, Faculdade de Medicina, Universidade de Sao Paulo, São Paulo, Brazil; 4 International Health Unit Vall d’Hebron-Drassanes, Infectious Diseases Department, Vall d’Hebron University Hospital, PROSICS Barcelona, Barcelona, Spain; 5 Centro de Investigación Biomédica en Red de Enfermedades Infecciosas (CIBERINFEC), Instituto de Salud Carlos III, Madrid, Spain; 6 Centre for Tropical Medicine, School of Medicine, University of Brasilia, Brasília, Distrito Federal, Brazil; 7 Laboratorio de Investigacao Medica em Parasitologia (LIM 46), Hospital das Clinicas, Faculdade de Medicina, Universidade de Sao Paulo, São Paulo, Brazil; 8 Nucleo de Parasitoses Sistemicas, Centro de Parasitologia e Micologia, Instituto Adolfo Lutz, São Paulo, Brazil; 9 Instituto de Infectologia Emilio Ribas, São Paulo, Brasil; 10 Vitalant Research Institute, San Francisco, California, United States of America; 11 Departamento de Medicina Preventiva, Faculdade de Medicina, Universidade de Sao Paulo, São Paulo, Brazil; The Ohio State University, UNITED STATES

## Abstract

**Background:**

*Trypanosoma cruzi* and HIV coinfection can evolve with depression of cellular immunity and increased parasitemia. We applied quantitative PCR (qPCR) as a marker for preemptive antiparasitic treatment to avoid fatal Chagas disease reactivation and analyzed the outcome of treated cases.

**Methodology:**

This mixed cross-sectional and longitudinal study included 171 Chagas disease patients, 60 coinfected with HIV. Of these 60 patients, ten showed Chagas disease reactivation, confirmed by parasites identified in the blood, cerebrospinal fluid, or tissues, 12 exhibited high parasitemia without reactivation, and 38 had low parasitemia and no reactivation.

**Results:**

We showed, for the first time, the success of the timely introduction of benznidazole in the non-reactivated group with high levels of parasitemia detected by qPCR and the absence of parasites in reactivated cases with at least 58 days of benznidazole. All HIV+ patients with or without reactivation had a 4.0–5.1 higher chance of having parasitemia than HIV seronegative cases. A positive correlation was found between parasites and viral loads. Remarkably, treated *T*. *cruzi/*HIV-coinfected patients had 77.3% conversion from positive to negative parasitemia compared to 19.1% of untreated patients. Additionally, untreated patients showed ~13.6 times higher Odds Ratio of having positive parasitemia in the follow-up period compared with treated patients. Treated and untreated patients showed no differences regarding the evolution of Chagas disease. The main factors associated with all-cause mortality were higher parasitemia, lower CD4 counts/μL, higher viral load, and absence of antiretroviral therapy.

**Conclusion:**

We recommend qPCR prospective monitoring of *T*. *cruzi* parasitemia in HIV+ coinfected patients and point out the value of pre-emptive therapy for those with high parasitemia. In parallel, early antiretroviral therapy introduction is advisable, aiming at viral load control, immune response restoration, and increasing survival. We also suggest an early antiparasitic treatment for all coinfected patients, followed by effectiveness analysis alongside antiretroviral therapy.

## Author summary

Chagas disease, present on all continents, in HIV-infected patients with cellular immunodeficiency may lead to the multiplication of *T*. *cruzi*. Reactivation of Chagas disease is life-threatening, occurring in immunosuppressed patients and diagnosed by parasite detection in blood or tissues. Here, we employed the quantitative Polymerase Chain Reaction (qPCR) to detect the increase of parasite copies in the blood and other biological fluids.

Twelve HIV-coinfected patients with increased parasitemia without reactivation and ten patients with Chagas disease reactivation were selected for antiparasitic treatment and compared with 38 HIV-coinfected untreated patients with low parasitemia. Antiparasitic treatment changed positive parasitemia to negative in 77.3% of treated patients, with or without reactivation, whereas 19% of untreated cases changed from positive to negative parasitemia, after 92 and 76 months of follow-up, respectively. Two patients with reactivation died before ten days of treatment, suggesting that the diagnosis and treatment were quite late. We recommend parasitemia monitoring by qPCR and point out the value of antiparasitic treatment to coinfected *T*. *cruzi*/HIV patients with high levels of parasites in the blood, alongside early antiretroviral introduction. We also suggest early antiparasitic treatment for all coinfected patients and the analysis of the role of benznidazole in the reduction of parasitemia and control of Chagas disease evolution.

## Introduction

Chagas disease affects 7–8 million people infected by the protozoan *T*. *cruzi*, mainly in Latin America [1]. *T*. *cruzi is* transmitted to humans via the excreta of blood-sucking infected triatomine bugs, ingestion of contaminated food, blood derivatives transfusion, congenital transmission, and organ transplant. Despite vector and blood transfusion control in endemic countries, it is now a health problem in several continents, due to increasing migratory flows of chronically infected populations to nonendemic areas [2–12]. Chagas disease is mostly found in the chronic phase, which follows an acute phase with high parasitemia. The chronic phase occurs with low and intermittent parasitemia, and without symptoms or organ involvement in ~70% of cases, but 20% to 40% of patients progress to the chronic cardiac or digestive forms [13,14].

*T*. *cruzi* /HIV coinfection occurs predominantly in Brazil and Argentina, but also in other countries such as the USA, Bolivia, Colombia, Chile, and Venezuela [15–18]. The prevalence of coinfection in persons living with HIV/AIDS ranges from 1.3% to 9.8%, with higher rates in drug users [17,19,20].

Under immunodepression induced by HIV, Chagas disease reactivation, an AIDS-defining condition [21,22], may emerge in chronically *T*. *cruzi* coinfected patients with CD4+ levels usually lower than 200 cells/μL, leading to high morbidity and case fatality higher than 70%. Reactivation rates range from 10% to 20% [17,23,24], with high rates reported in retrospective studies [17,25]. In addition, conclusive data about the interference of antiretroviral therapy on Chagas disease reactivation incidence are not available [23,26]. So, monitoring parasitemia in coinfected patients is essential for timely antiparasitic treatment aiming to decrease the parasitemia [27] and avoid Chagas disease reactivation [26,27]. Antiparasitic treatment has been previously recommended in case of detection of high parasitemia expressed by an increased rate of positive nymphs in xenodiagnosis [23]. Considering that parasitological enrichment methods have lower sensitivity, are time-consuming, and may be dangerous [14], the best options for parasitemia monitoring of infected patients under immunosuppression are molecular methods, mainly quantitative PCR [27–32].

Antiparasitic treatment is mandatory for acute Chagas disease and Chagas disease reactivation and has been recommended for all chronic Chagas disease patients under 50 years of age without severe cardiac or digestive impairment [13,33–39]. Concerning *T*. *cruzi*/HIV-coinfected patients, a small number of treated non-reactivated patients and short follow-up periods have been recorded, so there is no conclusive data on the effectiveness of antiparasitic treatment [13,15,18,40,41]. Based on previous data [23], the Brazilian Guidelines on Chagas disease recommend treatment of HIV- coinfected patients with high parasitemia as Class IIa, level of evidence C [13], whereas the Brazilian Society of Cardiology recommends treatment of these cases as Conditional grade, level B [42].

This study aims: 1. to analyze the role of qPCR for monitoring parasitemia in coinfected *T*. *cruzi*/HIV cases as a marker for preemptive therapy in patients without Chagas disease reactivation; 2. the use of qPCR to evaluate the efficacy of antiparasitic treatment, particularly in patients with high parasitemia and those with reactivation; and 3. to ascertain the evolution of Chagas disease and survival as outcomes of the treatment of coinfected patients.

A secondary objective of this study is to compare parasitemia before treatment in HIV + patients and HIV seronegative patients.

## Methods

### Ethics statement

The Institutional Review Board of the involved research centers approved the protocols (protocols number 095/1995 and 1043/07 at the coordinating center, Ethics and Research Committee of Hospital das Clinicas, Faculdade de Medicina, Universidade de Sao Paulo, São Paulo, Brazil). Informed written consent was obtained from patients prospectively enrolled. Using stored samples and medical charts of patients who dropped out of follow-up and were retrospectively included was approved by the Ethics Committee above, provided that confidentiality was respected.

### Patients

This mixed cross-sectional and longitudinal study included 171 patients with *T*. *cruzi* infection: 60 HIV-coinfected (HIV+) and 111 HIV seronegative (HIV negative), most of them recruited from the Infectious and Parasitic Diseases Division of the Hospital das Clinicas, a tertiary hospital attached to the School of Medicine of the University of São Paulo, Brazil. Fifty-two coinfected patients were consecutively enrolled from 1993 to 2020 at the AIDS Clinic of the same Division. Five patients were consecutively followed from 2008 to 2021 at Vall d’ Hebron University Hospital, Spain, two others from 1994 to 2007 at the Centre for Tropical Medicine, Brasilia, Brazil, and one from 2013 to 2019 at the Instituto de Infectologia Emílio Ribas, São Paulo, Brazil. Most patients were born in Brazil, except for three cases who were born in Bolivia, Ecuador, and El Salvador, respectively.

Benznidazole (5–7 mg/kg/d, for 60 days or until death) was prescribed for 22 of the 60 coinfected patients, of whom 10 showed Chagas disease reactivation (TR) and 12 did not (TNR). This treatment is mandatory for reactivated cases. For cases without reactivation but with high parasitemia, antiparasitic treatment was prescribed under medical advice due to the risk of Chagas disease reactivation [23], before publication of the Brazilian Consensus on Chagas Disease [13]. The remaining 38 patients with low parasitemia, were not treated. Clinical, epidemiological, and parasitological data of 12 treated patients and 32 untreated cases were previously partially published [23]. Clinical and cPCR data from two other untreated patients were also previously partially published [15].

In this paper, we report, for the first time, molecular (qPCR) results, parasitological and survival data for 20–254 months of follow-up post-antiparasitic treatment. We also included 38 untreated HIV-coinfected patients (UT) and 111 HIV seronegative patients with chronic Chagas disease (HIV-negative), as comparator groups.

We followed the participants for at least five years (or until death or drop out). HIV seronegative cases had annual clinical, cardiac (electrocardiogram, echocardiogram, and, if necessary, Holter), and digestive evaluations. HIV medical evaluations occurred every 3 to 6 months depending on their clinical and laboratory status, tolerance, adherence or resistance to antiretroviral therapy, and other comorbidities. Antiretrovirals, CD4, and HIV viral load were analyzed in the treated and untreated groups due to their possible influence on parasitemia before and after antiparasitic treatment.

All sociodemographic, clinical, and laboratory data, date of treatment and sample collections (before treatment and during follow-up), parasitemia results (parasitological and molecular methods), CD4 counts, HIV viral loads, antiretroviral treatment and schemes, the evolution of Chagas disease, all-cause mortality and deaths due to Chagas disease, were registered in the database [43].

#### Inclusion criteria

Patients included in the study were adults aged 18 years or more, who had been diagnosed with Chagas disease.

1. **Chagas disease** was diagnosed by the positive result of two out of three conventional serological tests with crude or recombinant *T*. *cruzi* antigens: indirect immunofluorescence (≥1/40 IIF), enzyme-linked immunosorbent assay (ELISA) and indirect hemagglutination (≥1/40 HA) [13].
2. **HIV infection** was determined with ELISA and confirmed by immunoblot [23].
3. **Chagas disease reactivation** was diagnosed by detection of *T*. *cruzi* in at least one of the following tests: direct blood microscopy or quantitative buffy coat (QBC), direct cerebrospinal fluid examination, or both. Clinical criteria for this investigation include signs of central or peripheral nervous system involvement, myocarditis, pericarditis, worsening of previous cardiomyopathy (arrhythmia, decrease in left ventricular ejection fraction, among others), panniculitis, fever, adenomegaly, hepatosplenomegaly, mononucleosis-like syndrome, mothers of premature newborns, with miscarriage or stillbirth, and oligosymptomatic patients in prospective follow-up periodically analyzed. For the clinical diagnosis of meningoencephalitis, imaging tests were performed (computed tomography or magnetic resonance imaging, and cerebrospinal fluid examination (cellularity, biochemistry, search for parasites using parasitological and molecular methods). If myocarditis or pericarditis were suspected, a conventional electrocardiogram or echocardiogram was performed. A biopsy was rarely indicated in meningoencephalitis or myocarditis but was used in panniculitis, followed by parasitological methods.

### Laboratory methods

#### Indirect parasitological assay

A blood culture assay was carried out as previously described [23]. Xenodiagnosis was performed with 20–40 nymphs of *Triatoma infestans* fed *in vitro* with 10 mL of the patient’s blood. Results are expressed as the percentage of positive insects (semiquantitative xenodiagnosis) or as a positive result if at least one insect was positive and a negative result if all insects were negative [23]. In two cases, *Dipetalogaster maximus* was employed and the results were expressed as positive or negative.

### Sample collection

Molecular analyses were performed retrospectively in most of the first samples and prospectively in most of the second samples. The last biological sample obtained before treatment (T0) and a sample collected about 5 years after the treatment (T1) were selected for comparison. Some patients died soon after starting the antiparasitic treatment and therefore had a short follow-up period. The database includes the T0-T1 intervals and each patient outcome [43]. For 10 non-reactivated HIV+ patients, more than five samples were collected before the antiparasitic treatment. The result of the last sample before treatment was considered for all comparative analyses except for the variable qPCR ≥200 par Eq/mL (parasite equivalent per milliliter of blood). If this high number of parasite Eq was detected in any sample of a patient, he/she was included among those with qPCR ≥200 par Eq/mL for analyses of Chagas disease evolution and death.

#### Sample preparation and DNA extraction

DNA was extracted from peripheral blood mononuclear cells collected in EDTA or 6M guanidine-HCl plus 0.2 M EDTA buffer (pH 8). From patients with central nervous system reactivation, DNA from the CSF was extracted from the supernatant and pellet, as previously reported [27].

#### Conventional PCR (cPCR)

cPCR was performed using the S35 and S36 primer pair, which amplifies a 330 bp minicircle sequence (Gibco Life Technologies) [44]. Two negative controls for the site of the master mix preparation and the site of DNA application, and one positive control consisting of 2×10^−15^ μg of Y strain of *T*. *cruzi* DNA were used. The presence of inhibitors was checked by amplification of duplicate patients´ samples containing a positive control of parasite DNA. The control samples were paired with patient samples to check for contamination.

#### Quantitative PCR assays (qPCR)

As previously reported, a 149 bp fragment was amplified by SYBR-Green-based real-time-PCR (qPCR) using primers TCZ3 and TCZ4 [27]. The standard amplification curve was prepared from a 10-fold dilution of DNA from blood spiked with 8x10^5^ to 8x10^-2^ par Eq/mL. In samples of five patients, a 166 bp fragment of genomic DNA was amplified by Real-Time PCR, with results expressed as detectable or undetectable, as previously recorded [3].

#### Criteria for antiparasitic treatment

1. Patients with Chagas disease reactivation, according to the criteria above described (number = 10);
2. Coinfected *T*. *cruzi*/HIV patients with high parasitemia (10 patients) by xenodiagnoses (≥20% of positivity on an individual examination of nymphs or ≥70% of positivity on tested pools). We included two other patients analyzed by Real-Time PCR, whose results were expressed as detectable or undetectable as described (3). Total number = 12 patients

#### Classification of clinical forms of chronic Chagas disease

Clinical forms were classified as: cardiac form with typical electrocardiographic changes or sinus bradycardia with <40 beats/min, right bundle-branch block, right bundle-branch + upper anterior fascicular blocks, second-degree or total atrioventricular block, and/or complex ventricular arrhythmia, T changes, electrically inactive area, sinus node dysfunction, non-sustained ventricular tachycardia); atypic cardiac form named as form with “significant non-specific electrocardiographic changes” [13], including sinus bradycardia (> 40 bpm), low voltage, incomplete right bundle-branch block, upper anterior fascicular block, first-degree atrioventricular block, and non-specific ST-T changes; indeterminate form with no symptoms or signs and normal electrocardiogram (ECG) and chest X-ray; digestive form with mega-esophagus or megacolon, and cardiac and digestive form.

#### Outcomes

The following outcomes were analyzed:

1. survival; and
2. the evolution of Chagas disease to a) improvement or unchanged clinical form versus b) increasing severity of the same clinical form (more severe cardiac or digestive forms, new arrhythmias, heart failure, complications of megacolon, or death due to Chagas disease).

We also examined the association of parasitemia (qPCR, qPCR ≥200 par Eq/mL, or all molecular and parasitological methods), CD4, viral load, and antiretroviral therapy with the clinical evolution of Chagas disease and survival.

### Statistical analysis

Analysis of sociodemographic, clinical, and molecular/parasitological traits pre-treatment with benznidazole was performed using the chi-square test and Fisher’s exact test for categorical variables. The Mann-Whitney U test was used to compare differences in continuous variables with non-parametric distribution between two independent groups. The T-test for independent samples was applied when the dependent variable was normally distributed. Paired samples were compared by Wilcoxon signed-rank test (paired samples Wilcoxon test). Initially, univariable analysis was performed to examine the unadjusted association between variables and outcomes. Logistic regression models were used to test the association of predictors with binary outcomes adjusted for potential confounders. The results from all logistic regression models were expressed as odds ratios (OR) with their corresponding 95% confidence interval (CI). Spearman’s rank correlation coefficient was used to test quantitative outcome variables not normally distributed (p< 0.05) [45]. McNemar’s test for two related dichotomous variables was used to examine the pre- and post-treatment results of the same patient. Differences in survival until the second blood collection (T1) between untreated cases, treated non-reactivated, and treated-reactivated patients were verified with the Kaplan-Meier curve and the log-rank test. All analyses were carried out with the statistical package SPSS (version 24.0, IBM, New York, NY, USA). Missing data and loss of follow-up were not considered in the analysis.

## Results

### HIV+ versus HIV seronegative patients at the first sample (T0)

In the present analysis, 171 patients with chronic Chagas disease were evaluated; among them, 60 patients were coinfected with HIV, of whom 22 (36.7%) were later submitted to treatment with benznidazole (Table 1).

**Table 1:** Patient sociodemographic traits, clinical characteristics of Chagas disease, and parasitemia levels according to HIV status.

Variable	HIV- (Ni = 111)	HIV+ (Ni = 60)	Statistical Analysis
**Age (years)**	**N = 111**	**N = 58**	**Mann-Whitney Test**
Median IQR (25–75%)	44.8 (37.8–59.4)	40.1 (33.7–52.7)	**p = 0.029**
**Sex** % (n/N)			**Chi-square test**
Male	44.1 (49/111)	56.7 (34/60)	p = 0.118
**White ethnicity** % (n/N)			**Chi-square test**
Yes	69.0 (69/100)	67.8 (40/59)	p = 0.505
**Birthplace (Region)** % (n/N)			**Fisher’s exact test**
North East	51.9 (54/104)	50.9 (29/57)	p = 0.112
South East	41.3 (43/104)	29.8 (17/57)	
South	2.9 (3/104)	7.0 (4/57)	
Other Regions + Other Countries	3.8 (4/104)	12.3 (7/57)	
**Clinical CD presentation** % (n/N)			**Fisher’s exact test**
Indeterminate clinical form (IF)	32.6 (29/89)	44.1 (26/59)	**p = 0.003**
Cardiac Form (CF)	21.3 (19/89)	28.8 (17/59)	
Digestive Form (DF)	25.8 (23/89)	11.9 (7/59)	
Cardiac and Digestive form (CDF)	7.9 (7/89)	15.3 (9/59)	
Non-specific ECG changes	12.4 (11/89)	0.0 (0/59)	
**Cardiac involvement** % (n/N)			**Chi-square test**
Yes % (n/N)	29.2 (26/89)	44.1 (26/59)	p = 0.064
**Indirect parasitological methods** % (n/N)			**Chi-square test**
Positive	19.4 (19/98)	65.3 (32/49)	**p<0.001**
**cPCR in blood** % (n/N)			**Chi-square test**
Positive	37.8 (42/111)	75.9 (41/54)	**p<0.001**
**Parasitemia in blood** % (n/N)			**Chi-square test**
Positive % (n/N)	41.4 (46/111)	78.3 (47/60)	**p<0.001**
**qPCR par Eq/mL of blood**	(N = 109)	(N = 48)	**Mann-Whitney Test**
Median (IQR 25–75%)	0.0 (0.0–0.1)	0.02 (0.0–13.3)	**p<0.001**
Minimum-Maximum	0.0–67.8	0.0–2585.0	

HIV- (negative) x HIV + (untreated, with and without Chagas disease reactivation patients); Ni: total included cases; N: number of analyzed cases for each variable; IQR: Interquartile range; Cardiac involvement: Cardiac and Cardiac + Digestive forms; Indirect parasitological methods: xenodiagnosis or/and blood culture; cPCR: Conventional PCR; Parasitemia in blood: xenodiagnosis, blood culture or/and cPCR; qPCR: quantitative PCR. Missing data are represented by the difference between the number of included patients in the first line (Ni) and the total number analyzed for each variable (N).

Table 1 shows the sociodemographic and clinical characteristics of HIV+ and HIV seronegative patients at the first collection. The HIV+ group had a lower age (p = 0.029). The statistical difference among clinical forms is attributed to the absence of “Significant non-specific ECG changes in HIV+ patients. Patients with cardiac involvement (Cardiac or Cardiac + Digestive) were more frequent HIV seropositive patients, although without statistical significance (Table 1). Parasitemia was more frequent in all HIV+ patients compared to HIV seronegative, in all methods used: indirect parasitological enrichment methods (blood culture, xenodiagnosis, or both), cPCR, or parasitemia (considering parasitological methods and/or cPCR, Table 1). In addition, higher levels of par Eq/mL were observed in the HIV + group (Table 1), compared to HIV seronegative.

### HIV+ without reactivation versus HIV seronegative patients at the first sample (T0)

To check whether parasitemia continues to be greater excluding the reactivation, considering that reactivated patients usually do not seek care in outpatient clinics, 50 coinfected patients without reactivation are compared to HIV seronegative patients. No differences were shown in age or clinical presentation (S1 Table), but greater parasitemia in the first group regarding parasitemia in the blood (parasitological methods and cPCR) by Chi-square Test p < 0.01, and qPCR (par Eq/mL by Mann-Whitney test p<0.024.

Multivariable logistic regression analysis showed that all HIV+ patients as a whole and HIV + patients without reactivation exhibited higher odds ratio (OR) of having *T*. *cruzi* detected by cPCR, indirect parasitological methods (blood culture and/or xenodiagnosis), or both than HIV seronegative patients, after adjustment for sex, ethnicity, age, and clinical form (S2 Table).

### Untreated (UT) HIV+ versus HIV-negative at the first sample (T0)

In a cross-section comparison of the group of patients not treated with benznidazole (UT) HIV+ and HIV seronegative patients, there were no differences between groups regarding sociodemographic or clinical variables (S1 Table).

Parasitemia was detected by parasitological and/or conventional molecular tests (cPCR) more frequently in untreated HIV+ patients than HIV seronegative patients. In contrast, the levels of par Eq/mL of blood by qPCR were similar (S1 Table).

Although HIV+ untreated patients had a higher odds ratio (OR) of having *T*. *cruzi* detected compared with HIV seronegative patients, this association was no longer significant in the multivariate analysis adjusting for age, sex, ethnicity, and clinical form (S3 Table).

Missing data before the treatment were attributed to technical reasons, absence of blood samples or only cerebrospinal fluid samples, and lack of information in medical records.

### Untreated (UT) HIV+ versus Treated (T) HIV + T0 at the first sample (T0)

The analysis comparing sociodemographic and clinical variables of HIV+ treated and untreated patients at the first collection showed no differences except for age. Treated patients were younger (Table 2).

**Table 2:** Characteristics of HIV+ patients untreated (UT) and treated (T) for Chagas disease before the antiparasitic treatment.

Variable	HIV + (Ni)				Statistical analysis
	^1^UT (Ni = 38)	^2^T (Ni = 22)	^3^TNR (Ni = 12)	^4^TR (Ni = 10)	
**Age (years)**	N = 37	N = 21	N = 11	N = 10	**Mann-Whitney Test**
Median (IQR 25%-75%)	46.2 (36.0–57.3)	35.5 (32.3–43.0)	37.2 (33.5–44.7)	33.7 (28.5–42.4)	^**1x2**^**p = 0.004;** ^**1x3**^**p = 0.110;** ^**3x4**^**p = 0.205**
**Sex**					**Chi-square test**
Male % (n/N)	52.6 (20/38)	63.6 (14/22)	66.7 (8/12)		^**1x2**^p = 0.407
				60.0 (6/10)	**Fisher’s exact test**
					^**1x3**^p = 0.510; ^**3x4**^p = 1.00
**White ethnicity**					**Fisher’s exact test**
Yes % (n/N)	64.9 (24/37)	72.7 (16/22)	66.7 (8/12)	80.0 (8/10)	^**1x2**^p = 0.532; ^**1x3**^**p = 0.013;**
					^3x4^p = 0.650
**Birthplace (Region) % (n/N)**					**Fisher’s exact test**
North East	54.3 (19/35)	45.5 (10/22)	41.7 (5/12)	50.0 (5/10)	^**1x2**^p = 0.711
South East	28.6 (9/35)	31.8 (7/22)	41.7 (5/12)	20.0 (2/10)	^**1x3**^p = 0.721
South	5.7 (2/35)	9.1 (2/22)	0.0 (0/12)	20.0 (2/10)	^**3x4**^p = 0.235
Other Regions + Other Countries	11.4 (4/35)	13.6 (3/22)	16.7 (2/12)	10.0 (1/10)	
**Cardiac involvement**					**Chi-square test**
Yes % (n/N)	37.8 (14/37)	54.5 (12/22)	50.0 (6/12)	60.0 (6/10)	^**1x2**^p = 0.456; ^**1x3**^p = 0.211;
					**Fisher’s exact test**
					^**3x4**^p = 0.691
**Indirect parasitological test**					**Fisher Exact Test**
Positive % (n/N)	43.3 (13/30)	100.0 (19/19)	100.0 (10/10)	100.0(9/9)	^**1x2**^**p<0.001;** ^**1x3**^**p = 0.002;** ^**3x4**^**NA**
**cPCR (blood)**					**Fisher’s exact test**
Positive % (n/N)	65.8 (25/38)	100.0 (16/16)	100.0 (9/9)	100.0(7/7)	^**1x2**^**p = 0.006;** ^**1x3**^**p = 0.047;** ^**3x4**^**NA**
**Parasitemia in blood**					**Fisher’s exact test**
Positive % (n/N)	65.8 (25/38)	100.0 (22/22)	100.0 (12/12)	100.0(10/10)	^**1x2**^**p = 0.001;** ^**1x3**^**p = 0.022;** ^**3x4**^**NA**
**Variable**	**HIV + (Ni)**				**Statistical analysis**
	^**1**^ **UT (Ni = 38)**	^**2**^ **T (Ni = 22)**	^**3**^ **TNR (Ni = 12)**	^**4**^ **TR (Ni = 10)**	
**qPCR par Eq/mL (blood)**	N = 34	N = 14	N = 7	N = 7	**Mann-Whitney Test**
Median (IQR 25%-75%)	0.0 (0.0–0.7)	275.1 (2.8–736.5)	3.7 (0.05–209.9)	594.0 (340.4–2585.0)	^**1x2**^**p<0.001;** ^**1x3**^**p = 0.014;**
Minimum-Maximum	0.0–67.8	0.0–2692.8	0.0–279.2	33.2–2692.8	^**3x4**^ **p = 0.006**
**CD4 count cells/μl**	N = 36	N = 20	N = 11	N = 9	**Mann-Whitney Test**
Median (IQR 25%-75%)	329.0 (138.7–485.0)	300.0 (35.3–564.0)	510.0 (140.0–787.0)	26.0 (10.0–317.5)	^**1x2**^**p = 0.431;** ^**1x3**^**p = 0.199;**
Minimum-Maximum	3–1823	4–1270	63–1270	4.0–406.0	^**3x4**^ **p = 0.009**
**CD4** ^**+**^					**Chi-square test**
< 200% (n/N)	36.1 (13/36)	45.0 (9/20)	27.3 (3/11)	66.7(6/9)	^**1x2**^p = 0.514;
					**Fisher’s exact test**
					^**1x3**^p = 0.725; ^**3x4**^p = 0.175
**VL count RNA viral copies/μL**	N = 30	N = 17	N = 11	N = 6	**Mann-Whitney Test**
Median	0.0	16124.0	1060.0	39499.5	^**1x2**^ **p = 0.021;**
IQR 25%-75%	0.0–3414.8	0.0–55466.0	0.0–32219.0	19537.5–3087500.0	^**1x3**^p = 0.204;
Minimum-Maximum	0.0–418000	0.0–11000000	0.0–750000	0.0–11000000	^**3x4**^p = 0.111
**VL detection**					**Chi-square test**
Detected % (n/N)	33.3 (10/30)	64.7 (11/17)	54.5 (6/11)		^**1x2**^ **p = 0.038**
				83.3 (5/6)	**Fisher’s exact test**
					^**1x3**^p = 0.287; ^**3x4**^p = 0.333
**ART**					**Fisher’s exact test**
No % (n/N)	40.5 (15/37)	81.8 (18/22)	75.0 (9/12)	90.0 (9/10)	^**1x2**^**p = 0.003;** ^**1x3**^p = 0.051; ^**3x4**^p = 0.594

Ni: total included cases; N: number of analyzed cases for each variable; UT: Non-treated patients; T: Treated patients (with and without Chagas disease reactivation patients); TNR: Treated patients (without Chagas disease reactivation patients); TR: Treated with Chagas disease reactivation patients; IQR: Interquartile range; Cardiac involvement: Cardiac and Cardiac + Digestive forms; Indirect parasitological methods: xenodiagnosis or/and blood culture; cPCR: conventional PCR; Parasitemia in blood: xenodiagnosis, blood culture or/and cPCR; qPCR: quantitative PCR; VL: Viral load; ART: Antiretroviral therapy. Missing data are represented by the difference between the number of included patients in the first line (Ni) and the total number analyzed for each variable (N).

Significant differences between groups were demonstrated with the detection of *T*. *cruzi* by indirect methods and cPCR, and higher levels by qPCR in both treated (T) and in the treated (T) than in the untreated group (UT).

Higher viral load and lower adherence to antiretroviral therapy were registered in the treated group (Table 2). Multivariate logistic regression analysis (Treated and Untreated groups) did not show an association between parasitemia and CD4 or viral load adjusted by age, sex, ethnicity, clinical form, and antiretroviral therapy (S4 Table).

However, we found a positive correlation between par Eq/mL (qPCR) and viral load (ρ = 0.366, p = 0.021, Spearman’s rank correlation coefficient) but not between par Eq/mL and CD4 level (p = 0.154, p = 0.312) using data on 45 out of 60 patients.

Similar results of parasitemia by parasitological, cPCR, and qPCR were observed when reactivated cases were excluded and treated non-reactivated patients were compared to untreated patients. Sociodemographic, clinical, or other laboratory differences were not shown (Table 2).

### Treated (T) HIV+ patients: reactivated versus non-reactivated (TNR) at T0

Patients with Chagas disease reactivation were younger than those in the non-reactivated group but this difference was not significant statistically (Table 2).

A higher number of parasites in the blood by qPCR (Table 2) and lower CD4 levels were seen in reactivated cases (Table 2). HIV viral load was also higher in this group but without a significant difference.

Direct parasitological tests were positive only in the reactivated patients, four in the blood and six in the cerebrospinal fluid. The clinical presentation at reactivation was meningoencephalitis in five cases, myocarditis in two, meningoencephalitis and myelitis in one, and two were oligosymptomatic.

Eight out of ten HIV+ patients showed clinical and laboratory evidence of Chagas disease reactivation at their first assessment on hospital admission and were not taking regular antiretroviral treatment.

Remarkably, patients with Chagas disease reactivation showed the highest levels of parasites detected by qPCR in the blood, before the treatment.

**Fig 1:**
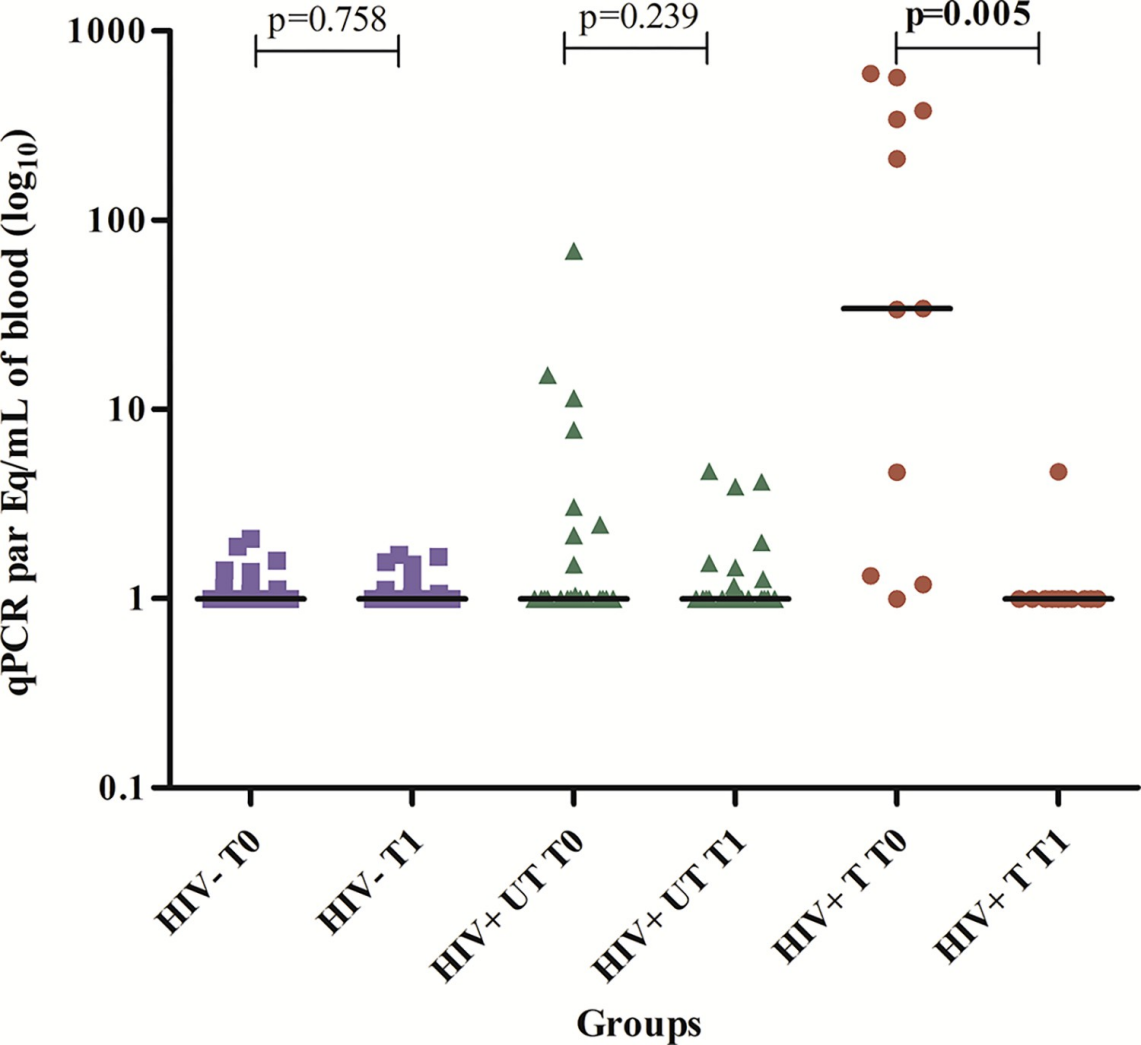
Parasitemia by qPCR in the first and second samples of HIV seronegative, HIV+ untreated (UT), HIV+ treated (T) non-reactivated + reactivated groups in the first and second samples.

Fig 1 shows a higher reduction of the levels of qPCR parasitemia levels of each treated patient after the treatment (T1) compared to his/her respective level before the treatment (T0) (Wilcoxon, p = 0.005).

Among the twelve treated patients without CD reactivation, only three were on antiretroviral therapy, had CD4 T cell counts greater than 500 cells/μL, and had undetectable viral loads. Despite these stable CD4 and viral load values, these three patients had positive parasitemia by cPCR and blood culture or xenodiagnosis in all samples collected before treatment with benznidazole.

### Parasitemia in the follow-up period

To evaluate the effect of benznidazole treatment, parasitemia in the post-treatment period was compared to the pre-treatment period. Untreated HIV+ patients with positive parasitemia at T0 (N+ **=** 21) were 13.6 times as likely to have positive parasitemia at re-sampling (T1: N+ = 17/21) than treated HIV+ patients (T0: N+ = 22; T1: N+ = 5/22) [OR = 13.60 (95% CI 3.09–59.83); p = 0.001].

**Fig 2:**
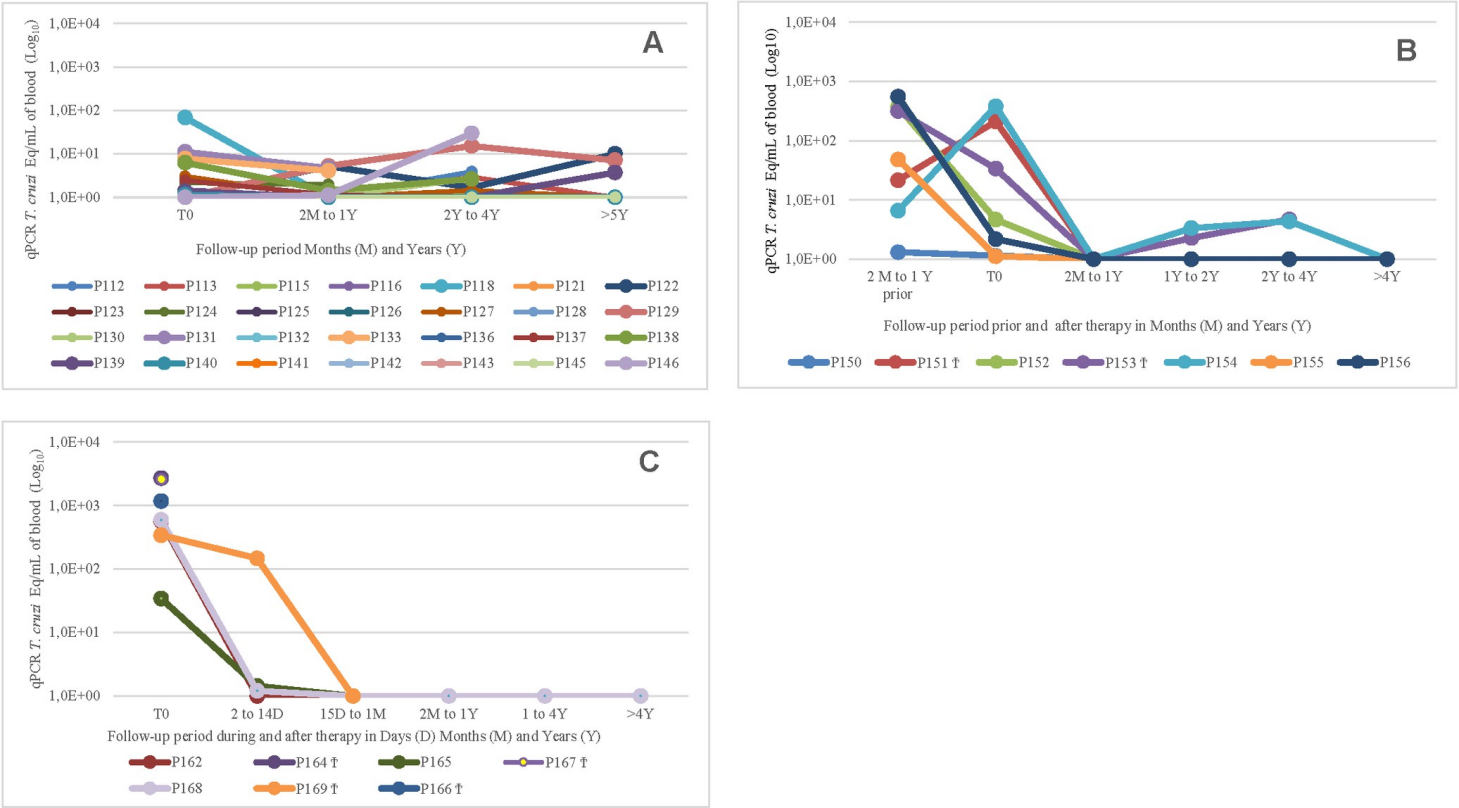
Evolution of qPCR (*T*. *cru*zi Eq/mL) in blood samples before and during the follow-up in treated and untreated patients. **A)** Untreated patients (UT)–no significant reduction of parasitemia during the period; **B**) Treated non-reactivated patients showing reduction of pre-treatment high levels. Some patients had high levels 2–12 months before the treatment which decreased at the beginning of treatment (T0); **C**: Treated and reactivated patients showed very high levels of parasitemia before the treatment and reduction of parasitemia.

In parallel, Fig 2 represents examples of the evolution of qPCR before, during, and after the treatment in treated non-reactivated and reactivated HIV+ groups compared to samples collected before and during the follow-up period in untreated HIV+ patients.

All 12 treated non-reactivated patients showed a reduction of parasitemia in the follow-up after the treatment. Nine out of ten showed undetectable levels of par Eq/mL by qPCR; one, only 3.7 par Eq/mL. The remaining two showed positive xenodiagnosis (qPCR not available). In eight of ten reactivated cases, no parasites were found in the blood, cerebrospinal fluid, or tissues after the first month of benznidazole treatment or in the follow-up period. Considering the 20 treated patients who survived four weeks after the end of the treatment, 85% had undetectable parasitemia in the second sample. In addition, even including two patients who died of Chagas disease reactivation, the rate of undetectable parasitemia remained high (Table 3 and Fig 2).

**Table 3:** Characteristics of HIV+ untreated (UT) and treated (T) patients with (T) and without (TNR) Chagas disease reactivation in the follow-up (T1).

Variable	HIV +			Statistical analysis
	^1^UT (Ni = 38)	^2^T (Ni = 22)	^3^TNR (Ni = 12)	
**CD Evolution** % (n/N)				**Chi-square test**
Clinical worsening	41.9 (13/31)	59.1 (13/22)	50.0 (6/12)	^**1x2**^p = 0.218; ^**1x3**^p = 0.633
**Death** % (n/N)				**Fisher’s exact test**
Chagas Disease	5.4 (2/37)	18.2 (4/22)	0.0 (0/12)	^**1x2**^p = 0.163
Others	13.5 (5/37)	22.7 (5/22)	33.3 (4/12)	^**1x3**^p = 0.241
**Period between T0 and T1 (months)**	N = 29	N = 22	N = 12	**Mann-Whitney test**
Mean±SD	88.3±73.4	79.7±74.3	98.1±50.7	
Median	70.9	72.0	120.0	^**1x2**^p = 0.413
IQR 25%-75%	26.2–153.1	9.1–132.5	39.0–133.4	
Minimum-Maximum	3.0–224.6	0.2–255.0	20.8–174.3	^**1x3**^p = 0.621
**Indirect parasitological test T1**				**Fisher’s exact test**
Positive % (n/N)	31.6 (6/19)	29.4 (5/17)	30.0 (3/10)	^**1x2**^p = 1.000; ^**1x3**^**p** = 1.000
**cPCR (blood) T1**				**Fisher’s exact test**
Positive % (n/N)	64.3 (18/28)	11.1 (2/18)	10.0 (1/10)	^**1x2**^**p = 0.001;** ^**1x3**^**p = 0.008**
**Parasitemia in blood T1**				**Fisher’s exact test**
Positive % (n/N)	67.9 (19/28)	23.8 (5/21)	25.0 (3/12)	^**1x2**^**p = 0.002;** ^**1x3**^**p = 0.018**
**qPCR par Eq/mL in blood T1**	(n = 24)	(n = 15)	(n = 8)	**Mann-Whitney test**
Median	0.0	0.0	0.0	^**1x2**^ **p = 0.046**
IQR 25%-75%	0.0–0.5	0.0–0.0	0.0–0.0	
Minimum-Maximum	0.0–17.0	0.0–3.7	0.0–3.7	^**1x3**^p = 0.255
**Parasitemia T0 and T1**				**Fisher’s exact test**
PN % (n/N)	19.1 (4/21)	77.3 (17/22)	75.0 (9/12)	^**1x2**^ **p<0.001**
PP % (n/N)	81.0 (17/21)	22.7 (5/22)	25.0 (3/12)	^**1x3**^ **p = 0.003**
**CD4** count **cells/ μL T1**	N = 24	N = 16)	N = 10	**T-test**
Mean±SD	619.1±281.9	557.2±293.0	601.4±288.4	^**1x2**^p = 0.653
Minimum-Maximum	137–1214	26–1055	26–1018	^**1x3**^p **=** 0.870
**CD4 cells/ μL**				**Fisher’s exact test**
< 200 % (n)	4.2 (1/24)	12.5 (2/16)	10.0 (1/10)	^**1x2**^p = 0.553; ^**1x3**^p = 0.508
**VL** count **RNA** viral copies/μL **T1**	N = 22	N = 13	N = 9	**Mann-Whitney test**
Median	0.0	0.0	0.0	^**1x2**^p *=* 0.710
IQR 25%-75%	0.0–110.0	0.0–55893.5	124158.0	
Minimum-Maximum	0–16600	0.0–260000	0.0–260000	^**1x3**^p = 0.848
**VL detection T1**				**Fisher’s exact test**
Detected % (n/N)	27.3 (6/22)	23.1 (3/13)	22.2 (2/9)	^**1x2**^p = 1.000; ^**1x3**^p = 1.000
**ART T1**				**Fisher’s exact test**
Yes % (n/N)	69.2 (18/26)	76.9 (10/13)	77.8 (7/9)	^**1x2**^p = 0.719; ^**1x3**^p = 1.000

^1^UT: Non-treated patients; ^2^T: Treated patients; ^3^TNR: Treated patients, without CD reactivation; Ni: total included cases; N: number of analyzed cases for each variable; CD: Chagas disease; T0: ^1st^sample from untreated and pre-therapy in treated patients; T1: ^2nd^sample from untreated and pos-therapy in treated patients; IQR: Interquartile range; Indirect parasitological methods: xenodiagnosis or/and blood culture; cPCR: conventional PCR; Parasitemia in blood: xenodiagnosis, blood culture or/and cPCR; qPCR: quantitative PCR; PN: Positive T0 and Negative T1; PP: Positive T0 and T1; VL: Viral Load; ART: Antiretroviral therapy. Missing data are represented by the difference between the number of included patients in the first line (Ni) and the total number analyzed for each variable (N).

**Fig 3:**
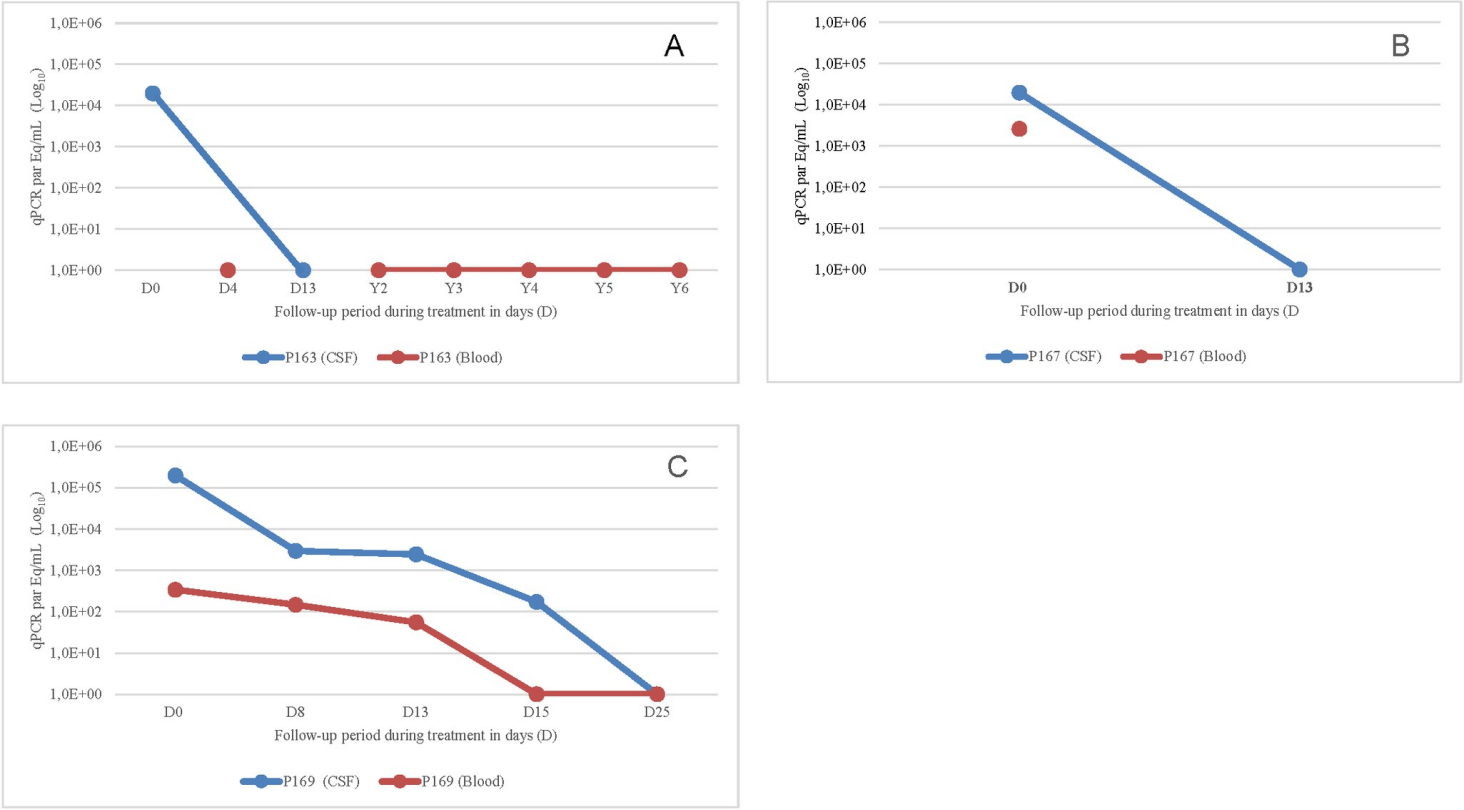
Comparison of qPCR (*T*. *cru*zi Eq/mL) in cerebrospinal fluid (CSF) and blood samples before and after the treatment in three patients. **A**) Patient resistant to antiretrovirals, with a viral load of 26,050 HIV copies/mL, 10 CD4 cells/μL, and in CSF, 19,530.0 par Eq/mL. After the antiparasitic treatment, undetectable levels of parasites in CSF and survival; **B)** Reduction of parasites but a moderate level was detected in the CSF before death; **C**) Reduction of parasites level to undetectable (blood, cerebrospinal fluid, and necropsy) after treatment, followed by death, possibly due to immune reconstitution syndrome, based on histopathologic findings, early ART introduction, and no confirmed causes of death.

In three cases with encephalitis, parasite levels were higher in cerebrospinal fluid (from 19,000 to 198,400 par Eq/mL) than in blood (412 to 2,586 par Eq/mL (Fig 3A–3C).

Table 3 shows significant differences in the evolution of parasitemia from T0 to T1 between treated and untreated groups by cPCR, qPCR, parasitemia (cPCR and/or parasitological tests), and evolution of parasitemia from positive to positive (PP), and positive to negative (PN). Seven untreated patients with undetectable parasitemia in T0 were excluded from this analysis. Analysis of the untreated HIV+ group showed that 81.0% (17/21) of those positive in the first sample remained positive in the second sample. No statistically significant difference was shown between the results of parasitemia (positive or negative) in the follow-up for this group (McNemar test, n = 28, p = 0.687).

Missing data in the follow-up after the treatment were attributed to technical reasons, absence of blood samples, lack of information in medical records, loss of follow-up (4/60 HIV+ patients), and deaths (16/60 HIV+ patients)

### Evolution of Chagas disease clinical forms

The evolution of Chagas disease to more severe forms was observed in both treated and untreated HIV+ patients during the follow-up (Table 3). Deaths due to Chagas disease occurred more frequently in the reactivated group, followed by untreated cases, and did not occur in the treated non-reactivated group, but no statistical difference was observed (Table 3).

Seven deaths occurred among the 38 untreated HIV+ patients during follow-up. Two died due to Chagas disease and the other five due to other causes (two—carcinoma, one—neuro-toxoplasmosis, one—sepsis, one—C hepatitis).

Nine deaths were observed among the treated HIV+ patients: five among those with reactivation and four in the non-reactivated group (Table 3).

Reactivation of Chagas disease was the main cause of death in four patients with meningoencephalitis. Two of them died after beginning treatment on the 5^th^ and 21^st^ day), no sample was available for the first and the second presented a considerable number of parasites in CSF during the treatment. Other two patients died on the 28^th^ and 56^th^ day of benznidazole without parasites detectable in the tissues. In one of them, no parasites were detected in blood and liquor either. Their deaths were attributed to other causes, sepsis or immune reconstitution (Fig 3C). So, although the reduction or disappearance of parasitemia was more frequently associated with survival, it does not rule out the evolution to death in patients with meningoencephalitis (Fig 3C). The cause of death in the fifth patient with reactivation was related to AIDS, two months after successful treatment with benznidazole.

Four deaths occurred in treated non-reactivated HIV+ patients during the follow-up period, none by Chagas disease, two due to AIDS-related causes, one due to heart attack, and the other, to sepsis.

**Fig 4:**
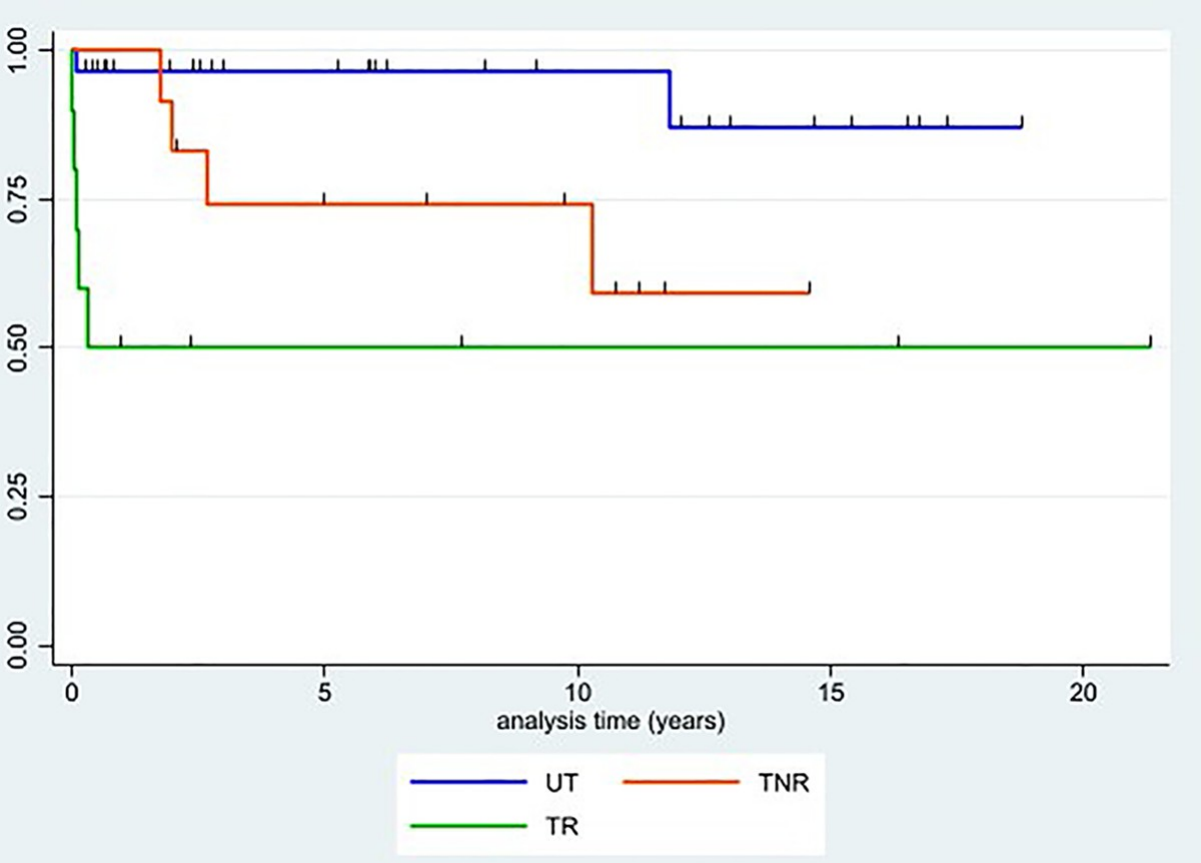
Kaplan-Meier survival estimates for untreated patients (UT), treated patients without Chagas disease reactivation (TNR) and treated patients who reactivated (TR). **Ticks on each curve represent censored individuals**.

Survival analysis of untreated, treated non-reactivated, and treated reactivated patients showed differences in death rates between the groups, particularly between untreated and treated reactivated (p = 0.001) (Fig 4). No significant differences were detected between treated non-reactivated and reactivated patients (p = 0.161), and untreated and treated non-reactivated (p = 0.034) curves, after applying a Bonferroni correction for multiple testing (p-value threshold 0.05/3 = 0.017).

Furthermore, considering all HIV + patients with *T*. *cruzi* infection, regardless of groups, the relationship between the evolution of Chagas disease and parasitemia (positive parasitological or molecular methods) and other variables was not statistically significant (Table 4).

**Table 4:** Evolution of Chagas disease clinical forms.

Variable	Clinical worsening		Statistical analysis
	No (n = 28)	Yes (n = 24)	
**Treated**			**Chi-square test**
Yes % (n/N)	33.3 (9/27)	50.0 (13/26)	p = 0.554
**Positive parasitemia**			**Chi-square test**
**T0**% (n/N)	77.8 (21/27)	88.5 (23/26)	p = 0.467
**T1**% (n/N)	45.8 (11/24)	52.0 (13/25)	p = 0.666
**qPCR par Eq/mL of blood**			**Mann-Whitney test**
**T0**	(n = 18)	(n = 23)	p = 0.222
Median IQR (25%-75%)	0.02 (0.0–7.7)	1.20 (0.0–340.4)	
Minimum-Maximum	0.0–567.1	0.0–2692.8	
**T1**	(n = 19)	(n = 19)	
Median IQR (25%-75%)	0.0 (0.0–0.5)	0.0 (0.0–0.0)	p = 0.147
Minimum-Maximum	0.0–17.0	0.0–3.7	
**qPCR≥200 par Eq/mL of blood**			**Fisher’s exact test**
**T0**% (n/N)	16.7 (3/18)	39.1 (9/23)	p = 0.171
**T1**% (n/N)	NA	NA	NA
**CD4 count cells/μL**			**Mann-Whitney test**
**T0**	(n = 20)	(n = 20)	
Median IQR (25%-75%)	297.0 (129.0–530.5)	332.0 (116.2–432.5)	p = 0.920
Minimum-Maximum	10–1823	4–1270	
**T1**	(n = 20)	(n = 20)	**T-test**
Mean (±SD)	640.2±273.5	564.4±295.0	p = 0.405
Minimum-Maximum	26–1214	40–1055	
**CD4**^**+**^ **<200 cells/μL**			**Chi-square test**
**T0**% (n/N)	40.0 (10/25)	29.2 (7/24)	p = 0.426
			**Fisher’s exact test**
**T1**% (n/N)	5.0 (1/20)	10.0 (2/20)	p = 1.000
**VL count RNA viral copies/μL**			
**T0**	(n = 20)	(n = 22)	**Mann-Whitney test**
Median IQR (25%-75%)	0.0 (0.0–21960.0)	0.0 (0.0–18635.0)	p = 0.900
Minimum-Maximum	0–58101	0–11000000	
**T1**	(n = 17)	(n = 18)	
Median IQR (25%-75%)	0.0 (0.0–4131.5)	0.0 (0.0–0.0)	p = 0.394
Minimum-Maximum	0.0–248316	0.0–0.260000	
**VL detection**			**Chi-square**
**T0**% (n/N)	45.0 (9/20)	36.4 (8/22)	p = 0.569
**T1**% (n/N)	35.3 (6/17)	16.7 (3/18)	p = 0.264
**No ART**			**Chi-square test**
**T0**% (n/N)	44.4 (12/27)	44.0 (12/25)	p = 0.947
**T1**% (n/N)	38.1 (8/21)	16.7 (3/18)	**Fisher’s exact test**
			p = 0.171

Positive parasitemia: xenodiagnosis, blood culture or/and cPCR; T0: 1^st^ sample from untreated and pre-therapy in treated patients; T1: 2^nd^ sample from untreated and pos-therapy in treated patients; qPCR: quantitative PCR; NR: Treated patients, without Chagas disease reactivation; IQR: Interquartile Range; VL: Viral Load; ART: Antiretroviral therapy; NA: Not applicable. Missing data are represented by the difference between the number of included patients in the first line (Ni) and the total number analyzed for each variable (N).

On the other hand, factors associated with all-cause mortality were CD4 < 200 cells/ μL and HIV viral load at T0 and T1, and qPCR ≥200 par Eq/mL and absence of antiretroviral therapy at T0 only (Table 5).

**Table 5:** All-cause mortality according to selected factors.

Variable	Death		Statistical analysis
	No (Ni = 43)	Yes (Ni = 16)	
**Treated**			**Chi-square test**
Yes % (n/N)	30.2 (13/43)	56.3 (9/16)	**p = 0.066**
**Positive parasitemia**			**Fisher’s exact test**
**T0**% (n/N)	76.7 (33/43)	81.3 (13/16)	p = 1.000
**T1**% (n/N)	46.2 (18/39)	60.0 (6/10)	p = 0.496
**qPCR par Eq/mL of blood**			**Mann-Whitney test**
**T0**	(n = 33)	(n = 14)	
Median (IQR 25–75%)	0.03 (0.0–594.0)	0.3 (0.0–546.3)	p = 0.326
Minimum-Maximum	0.0–6.04	0.0–2692.8	
**T1**	(n = 32)	(n = 7)	
Median (IQR 25–75%)	0.0 (0.0–0.2)	0.0 (0.0–0.0)	p = 0.535
Minimum-Maximum	0.0–17.0	0.0–3.7	
**qPCR≥200 par Eq/mL of blood**			**Fisher’s exact test**
**T0**% (n/N)	15.6 (5/32)	50.0 (7/14)	**p = 0.027**
**T1**% (n/N)	NA	NA	NA
**CD4 count cells/μL**			
**T0**	(n = 41)	(n = 15)	**Mann-Whitney test**
Median (IQR 25–75%)	329.0 (144.0–509.5)	114.0 (26.0–406.0)	p = 0.054
Minimum-Maximum	10–1823	3–1270	
**T1**			**T-test**
Mean (±SD)	649.9±255.3	315.0±333.4	**p = 0.013**
Minimum-Maximum	137–1214	26–808	
**CD4**^**+**^ **<200 cells/μL**			**Fisher’s exact test**
**T0**% (n/N)	29.3 (12/41)	66.7 (10/15)	**p = 0.027**
**T1**% (n/N)	2.9 (1/35)	40.0 (2/5)	**p = 0.036**
**VL count RNA viral copies/μL**			**Mann-Whitney test**
**T0**	n = 36	n = 11	
Median (IQR 25–75%)	0.0 (0.0–9102.5)	32219.0 (0.0–418000.0)	**p = 0.039**
Minimum-Maximum	0–450000	0–11000000	
**T1**	n = 32	n = 3	
Median (IQR 25–75%)	0.0 (0.0–0.0)	248316.0 (0.0)	**p = 0.020**
Minimum-Maximum	0.0–111787	0–260000	
**VL detection**			**Fisher’s exact test**
**T0**% (n/N)	36.1 (13/36)	58.3 (7/12)	p = 0.198
**T1**% (n/N)	21.9 (7/32)	66.7 (2/3)	p = 0.156
**No ART**			**Fisher’s exact test**
T0% (n/N)	46.5 (20/43)	81.3 (13/16)	**p = 0.020**
**T1**% (n/N)	25.0 (9/36)	66.7 (2/3)	p = 0.187

Ni: total included cases; N: number of analyzed cases for each variable. Positive parasitemia: xenodiagnosis, blood culture or/and cPCR; T0: 1^st^ sample from untreated and pre-therapy in treated patients; T1: 2^nd^ sample in untreated and pos-therapy in treated patients; qPCR: quantitative PCR; TNR: Treated patients, without Chagas disease reactivation; IQR: Interquartile Range; VL: viral load; ART: Antiretroviral therapy; NA: Not applicable. Missing data are represented by the difference between the number of included patients in the first line (Ni) and the total number analyzed for each variable (N).

Missing data in tables in the follow-up after treatment are attributed to technical reasons, absence of blood samples collected, and loss of follow-up.

The analysis of all-cause mortality excluding patients with reactivation showed an association with median RNA viral copies/μL (T0, 9/41 patients, Mann-Whitney test, U = 68.000, p = 0.006). Regarding high parasitemia, excluding the reactivation, no association with the worsening of Chagas disease was shown (N = 41, Fisher´s exact test, χ2(1) = 0.810; p = 0.656).

Given the small sample sizes and the presence of outliers, multivariate logistic regression analyses for Chagas disease evolution and mortality were not performed because their results would not have been reliable.

The main results of the statistical analysis are summarized in Box 1, according to the groups, table, size sample, methods, and p-value.

**Box 1. Summary of Statistical analysis results**

**Table:** 

Tables	Variable	Groups	Patients	Statistical analysis	p-value
**T1**	**cPCR**	HIV—x HIV +	165	Chi-square test	<0.001
		HIV—x HIV +(without CDR)	158		<0.001
	**Parasitemia**	HIV—x HIV +	171	Chi-square test	<0.001
		HIV—x HIV + (without CDR)	161		<0.001
	**qPCR**	HIV—x HIV +	157	Mann-Whitney test	<0.001
		HIV—x HIV + (without CDR)	150		0.024
**S1 Table**	**cPCR**	HIV—x HIV + (only UT)	149	Chi-square test	0.003
	**Parasitemia**	HIV—x HIV + (only UT)	149	Chi-square test	0.009
**S2 Table**	**Parasitemia**	HIV—x HIV +	171	Logistic Regression	<0.001
		HIV—x HIV + (TNR)	161		<0.001
		HIV—x HIV + (age+sex+ethnicity+IF)	138	Logistic Regression	0.004
		HIV—x HIV + (TNR/age+sex+ethnicity+IF)	128		0.015
**T2**	**cPCR**	HIV+: UT x T	54	Fisher’s exact test	0.006
		HIV+: UT x TNR	47		0.047
	**Parasitemia**	HIV+: UT x T	60	Fisher’s exact test	0.001
		HIV+: UT x TNR	50		0.022
	**qPCR**	HIV+: UT x T	48	Mann-Whitney test	<0.001
		HIV+: UT x TNR	41		0.014
	**VL (RNA viral copies/μL)**	HIV+: UT x T	47	Mann-Whitney test	0.021
	**ART**	HIV+: UT x T	59	Fisher’s exact test	0.003
**T3**	**qPCR**	HIV+ T: TNR x TR	48	Mann-Whitney test	0.006
	**CD4 cells/ μL**	HIV+ T: TNR x TR	56	Mann-Whitney test	0.009
**T4**	**PP % (n/N)**	HIV+ (T0 and T1): UT x T	43	Fisher’s exact test	*<*0.001
	**PP % (n/N)**	HIV+ (T0 and T1): UT x TNR	33		0.003
**T5**	**qPCR≥200 par Eq/mL**	Death: YES x NO (T0)	46	Fisher’s exact test	0.027
	**CD4 cells/ μL**	Death: YES x NO (T1)	40	T-test	0.013
	**CD4 count cells/ μL ≤200**	Death: YES x NO (T0)	56	Fisher’s exact test	0.027
		Death: YES x NO (T1)	40		0.036
	**VL count RNA viral copies/μL**	Death: YES xNO (T0)	47	Mann-Whitney test	0.039
		Death: YES x NO (T1)	35		0.020
**Lines 501–503**		Death: YES x NO (T0) (TNR)	41	Mann-Whitney test	0.016
		Death: YES x NO (T1) (TNR)	31		0.020
**Lines 357–3588**		qPCR par Eq/mL of blood x VL count RNA viral copies/μL	45	Spearman’s test	0.021

cPCR: conventional PCR; HIV— = HIV seronegative; Parasitemia in blood: Xenodiagnosis, blood culture or/and cPCR; qPCR: quantitative PCR; VL: Viral Load; ART: Antiretroviral therapy; PP: Positive parasitemia on 1^st^ and 2^nd^ samples; without CDR: without Chagas disease reactivation patients; IF: Indeterminate form of Chagas disease; UT: Untreated patients; T: Treated patients (with Chagas disease reactivation patients); TNR: Treated patients (without Chagas disease reactivation patients); T0: 1^st^ sample from untreated and pre-therapy in treated patients; T1: 2^nd^ sample from untreated and pos-therapy in treated patients.

## Discussion

In this study, we showed for the first time, that treated non-reactivated and reactivated patients showed a significant parasitemia reduction compared to parasitemia-positive untreated HIV+ patients. qPCR was undetectable during follow-up in 9/10 tested treated non-reactivated patients and very low in the 10^th^ case. No deaths due to Chagas disease were observed in treated non-reactivated cases in the follow-up (median 120 months, IQR: 33.2–133.4), but early deaths caused by Chagas disease were observed in reactivated patients.

The analysis of all-cause mortality showed lower survival in reactivated patients, followed by non-reactivated treated patients and untreated patients. The factors associated with mortality were qPCR ≥200 par Eq/mL, absence of ART, CD4 < 200 cells/ μL, HIV viral load before treatment, and the latter two in the follow-up post-therapy.

In addition, we demonstrated for the first time, that all HIV+ patients and HIV + without reactivation had a 4.0–5.1 higher chance of having parasitemia than HIV seronegative cases (S2 Table), and confirmed the correlation between viral and parasite loads. We also showed that HIV+ patients were younger than HIV seronegative individuals and that both had a similar frequency of cardiac forms.

The success of the timely benznidazole introduction in controlling *T*. *cruzi* parasitemia in the non-reactivated group with high parasitemia is similar to or better than observed in chronic patients [33,46,47]. Our data suggested that antiparasitic treatment in the non-reactivated group prevented Chagas disease reactivation or death due to Chagas disease, although not the worsening of Chagas disease. However, the sample size was small, and this result needs to be confirmed.

The higher survival in untreated than treated cases and the similarity in the evolution of Chagas disease in both groups can be explained by the characteristics of treated cases as the moderate severity of well-defined clinical forms before the treatment, higher parasitemia, lower adherence to antiretroviral therapy, less preserved immunity, early deaths in reactivated cases despite benznidazole therapy and small sample size. Furthermore, treated cases died mainly of Aids opportunistic disease, in contrast to untreated cases with a lower fatality rate due to several causes. In reactivated cases, two patients died in the first three weeks, a period possibly insufficient for benznidazole to exert its activity in severe meningoencephalitis, and two died in association with other causes. These findings point out the need for early treatment of coinfected cases before defined clinical forms become established and the importance of antiretroviral therapy in HIV+ patients´ survival.

Our findings are similar to a previous study, where the benefit of benznidazole for clinical evolution was not shown [46], and disagree with other reports [35–37,39].

In addition, the higher chance of HIV+ patients having parasitemia compared to HIV seronegative patients suggests that HIV+ patients are at a higher risk of Chagas disease reactivation (S2 Table), similar to other studies [27,31], in which univariate analysis was performed with smaller sample size as compared to ours. Our result points out the importance of HIV prevention and control, especially at younger ages, who are more frequently affected by HIV.

Furthermore, the observed significant correlation between qPCR (par Eq/mL) and viral load (RNA copies/μL), is similar to a previous report [27], and to the association seen between both and all-cause mortality, suggesting that uncontrolled HIV infection and consequent impaired immune response leads to increased parasite load as shown *in T*. *cruzi* and leukemia virus murine infection [48]. No correlation with CD4 count was noticed, as opposed to previous studies [27,31], possibly because of the smaller sample sizes or the presence of nonfunctional CD4 cells.

Moreover, parasitemia would be expected to influence clinical presentation [49,50]. The absence of a significant difference between clinically defined forms is likely explained by the presence of low or undetectable parasitemia in the majority of coinfected patients, similar to HIV seronegative cases, the slow evolution of Chagas disease damage and the small size of the remaining groups.

Although we consider that qPCR has been useful as a marker of preemptive therapy, parasitemia monitoring started in these cases when well-defined moderate forms were already established. Therefore, the search for early markers of the evolution of clinical forms represents a challenge. MicroRNAs have been highlighted but not tested yet in human disease or immunocompromised individuals [51–53].

Among the strengths of the present study are the demonstration of the usefulness of PCR as a marker for preemptive therapy and the successful reduction of parasitemia by antiparasitic treatment in the group with high parasitemia, the comparative data on Chagas disease survival in treated and untreated HIV+ patients, and the higher chance of HIV+ patients without reactivation having *T*. *cruzi* parasitemia pointing out the risk of reactivation. In addition, we confirmed the correlation between the par Eq levels and HIV viral load in blood.

The fact that this is an observational study and not a randomized controlled trial is certainly a limitation, in addition to having heterogeneous groups regarding disease severity and parasitemia levels, the characteristics of reactivated cases whose inclusion was not under control. Sample sizes were small, however, this represents the largest observational study on coinfection treatment, thus emphasizing its underreporting. Furthermore, patients with low or undetectable parasitemia were not treated. As reported, the decision to treat patients with high parasitemia was made before the publication of a consensus recommending antiparasitic treatment for all chronic patients [13]. Finally, as qPCR was an in-house technique, several parameters may vary for comparison with other reports.

Considering the lack of data on the cost-benefit of antiparasitic therapy for all coinfected seropositive HIV patients and the role of both benznidazole and antiretroviral therapy shown in the present work, a large multicenter study would be useful to evaluate the effectiveness and the toxicity of antiparasitic treatment together with antiretroviral therapy in this group of patients. It is a challenge to know whether early treatment of coinfected patients reduces parasitemia in the same way and prevents the evolution to severe clinical forms.

## Conclusion

We recommend qPCR prospective monitoring of *T*. *cruzi* parasitemia in HIV+ patients and point out the value of pre-emptive therapy for patients with high parasitemia. In parallel to parasitemia monitoring, an early antiretroviral therapy introduction is advisable, aiming at viral load control and immune response restoration.

We also suggest an earlier antiparasitic treatment for all coinfected patients, followed by effectiveness analysis alongside antiretroviral therapy.

## Supporting information

S1 Table Untreated patient’s age, cardiac form, and parasitemia levels according to HIV status. (DOCX)

S2 Table Multivariate logistic regression for *T*. *cruzi* parasitemia detection in HIV+ (with and without Chagas disease reactivation), and HIV seronegative patients. (DOCX)

S3 Table Parasitemia vs HIV status in untreated HIV+ and HIV seronegative patients. Unadjusted and adjusted logistic regression.
(DOCX)

S4 Table Parasitemia (indirect parasitological and molecular methods) vs CD4 and viral load (VL) in HIV+ treated (T) and untreated (UT) patients. Unadjusted and adjusted logistic regression.
(DOCX)
