# Correction: Quantitative PCR as a marker for preemptive therapy and its role in therapeutic control in *Trypanosoma cruzi*/HIV coinfection

**DOI:** 10.1371/journal.pntd.0013712

**Published:** 2025-11-13

**Authors:** Vera Lúcia Teixeira de Freitas, Christina Terra Gallafrio Novaes, Ana Marli Christovam Sartori, Noemia Barbosa Carvalho, Sheila Cristina Vicente da Silva, Érika Shimoda Nakanishi, Fernando Salvador, Cleudson Nery de Castro, Rita Cristina Bezerra, Elizabeth Visone Nunes Westphalen, Caroline Medeji Ramos de Oliveira, Felipe Delatorre Busser, Yeh-Li Ho, Renata Buccheri, Carolina Bonilla, Maria Aparecida Shikanai-Yasuda

In Table 1, the value in the “Minimum-Maximum” of the variable “qpCR par Eq/mL of blood” under the HIV-(Ni = 111) column is incorrect. Please see the correct Table 1 below.

**Table 1:** Patient sociodemographic traits, clinical characteristics of Chagas disease, and parasitemia levels according to HIV status.

Variable	HIV- (Ni = 111)	HIV+ (Ni = 60)	Statistical Analysis
**Age (years)**	**N = 111**	**N = 58**	**Mann-Whitney Test**
Median IQR (25–75%)	44.8 (37.8 - 59.4)	40.1 (33.7-52.7)	***p* = 0.029**
**Sex** % (n/N)			**Chi-square test**
Male	44.1 (49/111)	56.7 (34/60)	*p* = 0.118
**White ethnicity** % (n/N)			**Chi-square test**
Yes	69.0 (69/100)	67.8 (40/59)	*p* = 0.505
**Birthplace (Region)** % (n/N)			**Fisher’s exact test**
North East	51.9 (54/104)	50.9 (29/57)	*p* = 0.112
South East	41.3 (43/104)	29.8 (17/57)	
South	2.9 (3/104)	7.0 (4/57)	
Other Regions + Other Countries	3.8 (4/104)	12.3 (7/57)	
**Clinical CD presentation** % (n/N)			**Fisher’s exact test**
Indeterminate clinical form (IF)	32.6 (29/89)	44.1 (26/59)	***p* = 0.003**
Cardiac Form (CF)	21.3 (19/89)	28.8 (17/59)	
Digestive Form (DF)	25.8 (23/89)	11.9 (7/59)	
Cardiac and Digestive form (CDF)	7.9 (7/89)	15.3 (9/59)	
Non-specific ECG changes	12.4 (11/89)	0.0 (0/59)	
**Cardiac involvement** % (n/N)			**Chi-square test**
Yes % (n/N)	29.2 (26/89)	44.1 (26/59)	p = 0.064
**Indirect parasitological methods** % (n/N)			**Chi-square test**
Positive	19.4 (19/98)	65.3 (32/49)	***p* < 0.001**
**cPCR in blood** % (n/N)			**Chi-square test**
Positive	37.8 (42/111)	75.9 (41/54)	***p* < 0.001**
**Parasitemia in blood** % (n/N)			**Chi-square test**
Positive % (n/N)	41.4 (46/111)	78.3 (47/60)	***p* < 0.001**
**qPCR par Eq/mL of blood**	(N = 10)	(N = 48)	**Mann-Whitney Test**
Median (IQR 25–75%)	0.0 (0.0-0.1)	0.02 (0.0-13.3)	***p* < 0.001**
Minimum-Maximum	0.0-10.8	0.0-2585.0	

HIV- (negative) x HIV + (untreated, with and without Chagas disease reactivation patients); Ni: total included cases; N: number of analyzed cases for each variable; IQR: Interquartile range; Cardiac involvement: Cardiac and Cardiac + Digestive forms; Indirect parasitological methods: xenodiagnosis or/and blood culture; cPCR: Conventional PCR; Parasitemia in blood: xenodiagnosis, blood culture or/and cPCR; qPCR: quantitative PCR. Missing data are represented by the difference between the number of included patients in the first line (Ni) and the total number analyzed for each variable (N).
