# Supplementary material for: Quantitative PCR as a marker for preemptive therapy and its role in therapeutic control in Trypanosoma cruzi/HIV coinfection
Source: PLoS Negl Trop Dis. 2024 Feb 26;18(2):e0011961. doi: 10.1371/journal.pntd.0011961 (PMC10896531; doi:10.1371/journal.pntd.0011961)
Supplement: S1 Table — (DOCX) [file pntd.0011961.s001.docx]

**S1 Table**. Untreated patient's age, cardiac form, and parasitemia levels according to

HIV status.

| **Variable** | **^1^HIV-** | **^2^HIV+ UT** | **^3^HIV+ UT+TNR** | **Statistical analysis** |
| --- | --- | --- | --- | --- |
|  | **Ni=111** | **Ni=38** | **Ni=50** |  |
| **Age (years)** | N=111 | N=37 | N=48 | **Mann-Witney Test** |
| Median (IQR 25-75%) | 44.8 (37.8-59.4) | 46.2 (236.0-57.3) | 43.8 (35.6-55.1) | **^1x2^**p=0.644; **^1x3^**p=0.216 |
| **Cardiac involvement** |  |  |  | **Chi-square test** |
| Yes % (n/N) | 29.2 (26/89) | 37.8 (14/37) | 40.8 (20/49) | **^1x2^**p=0.334; **^1x3^**p=0.166 |
| **Indirect parasitological methods** |  |  |  | **Chi-square test** |
| Positive % (n/N) | 19.4 (19/98) | 43.3 (13/30) | 57.5 (23/40) | **^1x2^p=0.008;^1x3^p<0.001** |
| **cPCR in blood** |  |  |  | **Chi-square test** |
| Positive % (n/N) | 37.8 (42/111) | 65.8 (25/38) | 72.3 (34/37) | **^1x2^p=0.003; ^1x3^p<0.001** |
| **Parasitemia in blood** |  |  |  | **Chi-square test** |
| Positive % (n/N) | 41.4 (46/111) | 65.8 (25/38) | 74.0 (37/50) | **^1x2^p=0.009; ^1x3^p<0.001** |
| **qPCR par Eq/mL of blood** | N=109 | N=34 | N=41 | **Mann-Witney Test** |
| Median (IQR 25-75%) | 0.0 (0.0-0.1) | 0.0 (0.0-0.7) | 0.0 (0.0-1.8) | **^1x2^**p=0.264;  **^1x3^**p=0.024 |
| Minimum-Maximum | 0.0-11.0 | 0.0-67.8 | 0.0-379.2 |  |

^1^HIV-: HIV seronegative, ^1^HIV+ UT: HIV + untreated; ^3^HIV+ UT+TNR - HIV+ untreated + non-reactivated; Ni: total number of included patients: N: number of analyzed patients; IQR: interquartile interval, Cardiac involvement: Cardiac and Cardiac + Digestive forms; Indirect parasitological methods: xenodiagnosis or/and blood culture; cPCR: conventional PCR; Parasitemia in blood: xenodiagnosis, blood culture or/and cPCR; qPCR: quantitative PCR. Missing data are represented by the difference between the number of included patients in the first line (Ni) and the total number analyzed for each variable (N).
