# Supplementary material for: Quantitative PCR as a marker for preemptive therapy and its role in therapeutic control in Trypanosoma cruzi/HIV coinfection
Source: PLoS Negl Trop Dis. 2024 Feb 26;18(2):e0011961. doi: 10.1371/journal.pntd.0011961 (PMC10896531; doi:10.1371/journal.pntd.0011961)
Supplement: S2 Table — (DOCX) [file pntd.0011961.s002.docx]

**S2 Table**. Multivariate logistic regression for *T. cruzi* parasitemia detection in HIV+ (with and without Chagas disease reactivation), and HIV seronegative patients.

|  | **N** | **OR** | **95% CI** | **p** |
| --- | --- | --- | --- | --- |
| **HIV+ (Parasitemia +)** | **Ni=171** | **5.109** | **2.484-10.506** | **<0.001** |
| **HIV+ (Parasitemia +)** | **N=138** | **3.077** | **1.392-6.801** | **0.005** |
| **HIV + (Age)** |  | 0.970 | 0.941-1.000 | 0.051 |
| **HIV + (Sex M)** |  | 0.579 | 0.272-1.230 | 0.155 |
| **HIV + (White Y)** |  | 1.084 | 0.490-2.399 | 0.842 |
| **HIV + (CF Y)** |  | 0.584 | 0.268-1.277 | 0.178 |
| **HIV+ without CDR (Parasitemia +)** | **Ni=161** | **4.022** | **1.926-8.397** | **<0.001** |
| **HIV+ (Parasitemia +)** | **N=128** | **2.590** | **1.160-5.782** | **0.020** |
| **HIV + (Age)** |  | 0.979 | 0.949-1.011 | 0.193 |
| **HIV + (Sex M)** |  | 1.791 | 0.824-3.896 | 0.141 |
| **HIV + (White Y)** |  | 0.816 | 0.363 -1.837 | 0.624 |
| **HIV + (CF Y)** |  | 1.557 | 0.691 -3.508 | 0.285 |

Ni: total number of included patients. N: number of patients for this analysis. OR: Odds ratio, 95% CI: 95% Confidence interval, M: Male; Y: Yes; CF: Cardiac Form; CDR: Chagas disease reactivation patients. Missing data are represented by the difference between the number of included patients in the first line (Ni) and the total number analyzed for each variable (N).
