# Supplementary material for: Quantitative PCR as a marker for preemptive therapy and its role in therapeutic control in Trypanosoma cruzi/HIV coinfection
Source: PLoS Negl Trop Dis. 2024 Feb 26;18(2):e0011961. doi: 10.1371/journal.pntd.0011961 (PMC10896531; doi:10.1371/journal.pntd.0011961)
Supplement: S3 Table — Unadjusted and adjusted logistic regression. (DOCX) [file pntd.0011961.s003.docx]

**S3 Table**. Parasitemia vs HIV status in untreated HIV+ and HIV seronegative patients. Unadjusted and adjusted logistic regression.

|  | **N** | **OR** | **95% CI** | **p** |
| --- | --- | --- | --- | --- |
| **HIV+** | Ni=149 | 2.717 | 1.259-5.865 | **0.011** |
| **HIV+ (Parasitemia +)** | N=117 | 1.965 | 0.857-4.506 | 0.110 |
| **HIV + (Age)** |  | 0.990 | 0.957-1.025 | 0.569 |
| **HIV + (Sex M)** |  | 1.785 | 0.787-4.046 | 0.165 |
| **HIV + (White Y)** |  | 0.829 | 0.352-1.948 | 0.666 |
| **HIV + (IF Y)** |  | 1.052 | 0.448-2.470 | 0.908 |

Ni: total number of included patients. N: number of patients for this analysis; OR: Odds Ratio; 95% CI: Confidence interval; p<0.005 is significant; M: Male, Y: Yes; IF: Indeterminate form. Missing data are represented by the difference between the number of included patients in the first line (Ni) and the total number analyzed for each variable (N).
