# Supplementary material for: Quantitative PCR as a marker for preemptive therapy and its role in therapeutic control in Trypanosoma cruzi/HIV coinfection
Source: PLoS Negl Trop Dis. 2024 Feb 26;18(2):e0011961. doi: 10.1371/journal.pntd.0011961 (PMC10896531; doi:10.1371/journal.pntd.0011961)
Supplement: S4 Table — Unadjusted and adjusted logistic regression. (DOCX) [file pntd.0011961.s004.docx]

**S4 Table**. Parasitemia (indirect parasitological and molecular methods) vs CD4 and viral load (VL) in HIV+ treated (T) and untreated (UT) patients. Unadjusted and adjusted logistic regression.

|  | **N** | **OR** | **95% CI** | **p** |
| --- | --- | --- | --- | --- |
| **CD4<200 (Y)** | Ni=56 | 1.448 | 0.476-4.405 | 0.515 |
| **CD4<200 (Y)** | N=55 | 1.696 | 0.432-6.657 | 0.449 |
| **Age (years)** |  | 0.941 | 0.879-1.008 | 0.085 |
| **Sex (M)** |  | 1.458 | 0.371-5.722 | 0.589 |
| **White (Y)** |  | 2.044 | 0.493-8.483 | 0.325 |
| **Indeterminate Form (Y)** |  | 0.587 | 0.149-2.305 | 0.445 |
| **ART (No)** |  | 4.330 | 1.012-18.529 | 0.048 |
| **VL (Detectable)** | Ni=48 | 3.000 | 0.881-10.210 | 0.079 |
| **VL (Detectable)** | N=47 | 2.278 | 0.551-9.421 | 0.256 |
| **Age (years)** |  | 0.919 | 0.919-0.989 | 0.024 |
| **Sex (M)** |  | 1.223 | 0.296-5.047 | 0.781 |
| **White (Y)** |  | 1.296 | 0.291-5.782 | 0.734 |
| **Indeterminate Form (Y)** |  | 1.549 | 0.367-6.539 | 0.551 |
| **CD4<200 (Y)** | Ni=48 | 1.000 | 0.272-0.671 | 1.000 |
| **VL (Detectable)** |  | 3.00 | 0.874-10.300 | 0.081 |

Ni: total number of included patients. N: number of patients for this analysis; OR: Odds ratio; 95% CI=Confidence interval, Y: Yes; M: Male; ART: Antiretroviral therapy. Missing data are represented by the difference between the number of included patients in the first line (Ni) and the total number analyzed for each variable (N).
